# Platelet-rich-fibrin: A novel root coverage approach

**DOI:** 10.4103/0972-124X.51897

**Published:** 2009

**Authors:** K. Anilkumar, A. Geetha, Umasudhakar, T Ramakrishnan, R Vijayalakshmi, E. Pameela

**Affiliations:** 1 *Lecturer, Department of Periodontics, Meenakshi Ammal Dental College, Madhuravoyal, Chennai – 600 095, India*; 2 *Lecturer, Department of Periodontics, Meenakshi Ammal Dental College, Madhuravoyal, Chennai – 600 095, India*; 3 *Professor, Department of Periodontics, Meenakshi Ammal Dental College, Madhuravoyal, Chennai – 600 095, India*; 4 *Professor, Department of Periodontics, Meenakshi Ammal Dental College, Madhuravoyal, Chennai – 600 095, India*; 5 *Lecturer, Department of Periodontics, Meenakshi Ammal Dental College, Madhuravoyal, Chennai – 600 095, India*; 6 *Professor and HOD, Department of Periodontics, Meenakshi Ammal Dental College, Madhuravoyal, Chennai – 600 095, India*

**Keywords:** Plasma rich-derivative (PRF membrane), recession, regeneration, repair

## Abstract

Treatment of gingival recession has become an important therapeutic issue due to increasing cosmetic demand. Multiple surgical procedures have been developed to obtain predictable esthetic root coverage. More specifically, after periodontal regenerative surgery, the aim is to achieve complete wound healing and regeneration of the periodontal unit. A recent innovation in dentistry is the preparation and use of platelet-rich plasma (PRP), a concentrated suspension of the growth factors, found in platelets. These growth factors are involved in wound healing and postulated as promoters of tissue regeneration. This paper reports the use of PRF membrane for root coverage on the labial surfaces of the mandibular anterior teeth. This was accomplished using laterally displaced flap technique with platelet rich fibrin (PRF) membrane at the recipient site.

## INTRODUCTION

Periodontal plastic surgery procedures aimed at coverage of exposed root surfaces have evolved into routine treatment modalities. The main indications for root coverage procedures are esthetic concern, root hypersensitivity, prevention or management of root caries and cervical abrasion, enhancement of restorative outcomes, and facilitation of plaque control efforts. Among them, traumatic tooth brushing and tooth mal-positioning have been related most frequently to gingival recession.[1] The goal of periodontal therapy is to protect and maintain the patient's natural dentition over his or her lifetime for optimal comfort, function and esthetic appearance.[2,3] Therefore, marginal gingival recession should not be viewed as merely a soft tissue defect, but rather as the destruction of both soft and hard tissue.

Since mid 20^th^ century, different techniques have been developed to cover denuded roots. Free autogenous grafts and pedicle grafts including rotational flaps, advanced flaps, and semilunar flaps have been advocated. Combination grafts with either autogenous grafts or allograft and with GTR membranes were developed later to correct mucogingival defects.[4]

A recent innovation in dentistry is the preparation and use of platelet-rich fibrin (PRF), a concentrated suspension of the growth factors found in platelets. These growth factors are involved in wound healing and are postulated as promoters of tissue regeneration.[5] Platelet concentrate contains PDGF, TGF and many other unidentified growth factors that modulate and upregulate one growth factors function in the presence of second or third growth factor.[6] This specific feature influenced the decision to use platelet concentrates as the test material of choice in this case report. In the case described in this article, platelet rich derivative (PRF membrane) was combined with a laterally positioned flap for root coverage.

### Clinical presentation

**Figure 1:**
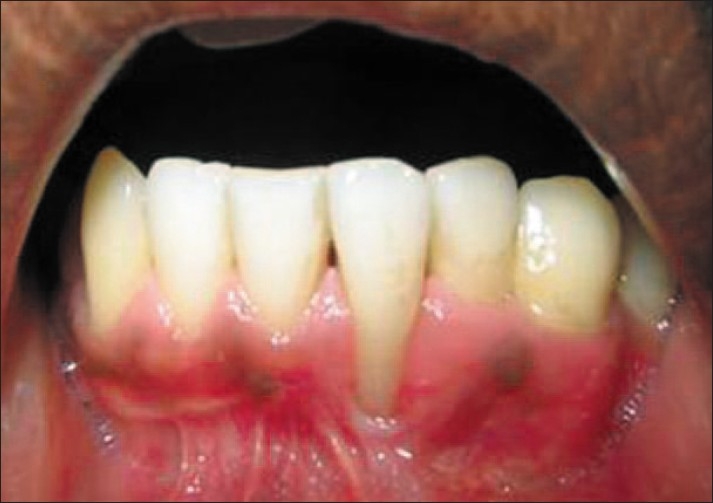
Pre-operative view

A 19-year-old male was referred by his general dentist for an evaluation of recession over the buccal prominence of the mandibular left incisor close to the mucogingival junction. At the time of presentation, clinical examination revealed 7 mm of clinical attachment loss. The distance between the cemento-enamel junction and gingival margin was 5 mm and the distance between the gingival margin and the base of the pocket was 2 mm [Figure 1].

#### Presurgical therapy:

The surgical procedure was explained to the patient and the informed consent obtained. Preparation of the patient included scaling and root planing of the entire dentition and oral hygiene instructions. The following parameters were recorded before and after surgery.

#### Probing pocket depth (PPD):

Gingival recession (GR), by measuring the distance between the cementoenameljunction (CEJ) to the free gingival margin.

### PRF preparation

The advantages of PRF over PRP are its simplified preparation and lack of biochemical handling of the blood. The required quantity of blood is drawn in 10 ml test tubes without an anticoagulant and centrifuged immediately. Blood is centrifuged using a tabletop centrifuge (REMY Laboratories) for 12 minutes at 2,700 rpm.[7]

**Figure 2:**
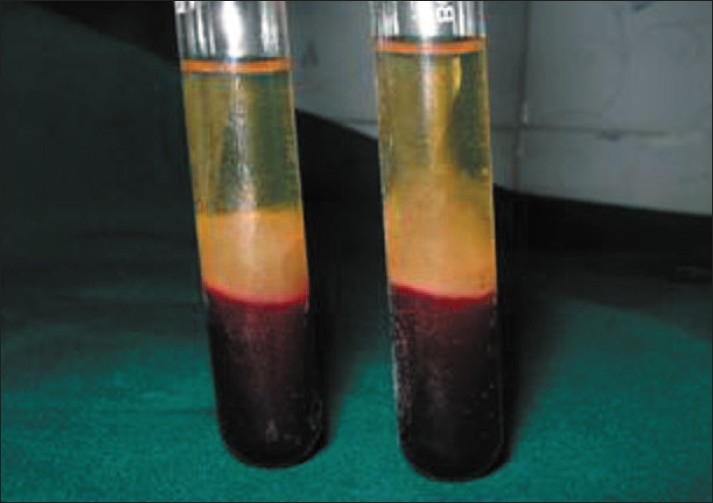
Platelet-rich-fibrin clot

The resultant product consists of the following three layers [Figure 2]:

- Top most layer consisting of acellular PPP
- PRF clot in the middle
- RBCs at the bottom

**Figure 3:**
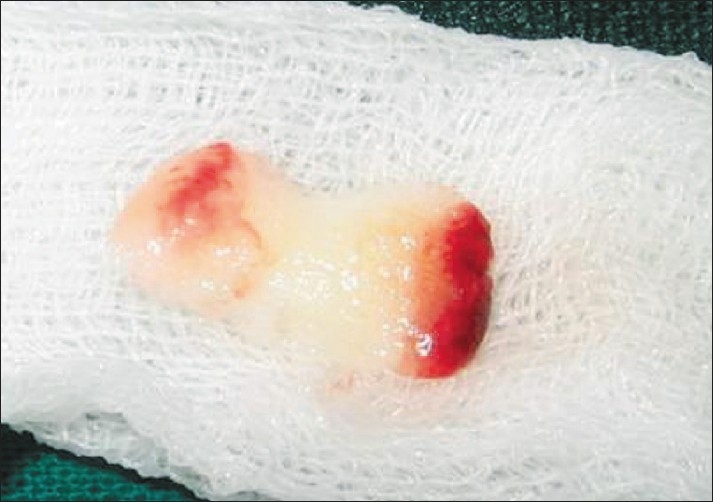
Platelet-rich-fibrin membrane

Because of the absence of an anticoagulant, blood begins to coagulate as soon as it comes in contact with the glass surface. Therefore, for successful preparation of PRF, speedy blood collection and immediate centrifugation before the clotting cascade is initiated, is absolutely essential. PRF can be obtained in the form of a membrane by squeezing out the fluids in the fibrin clot[7] [Figure 3].

#### Surgical procedure:

The use of laterally repositioned flap to cover areas with localized recession was introduced by Grupe and Warren in 1956. This technique involved the reflection of a full thickness flap/partial thickness flap at the donor area adjacent to the defect and subsequent displacement of this flap to cover the exposed root surface.

**Figure 4:**
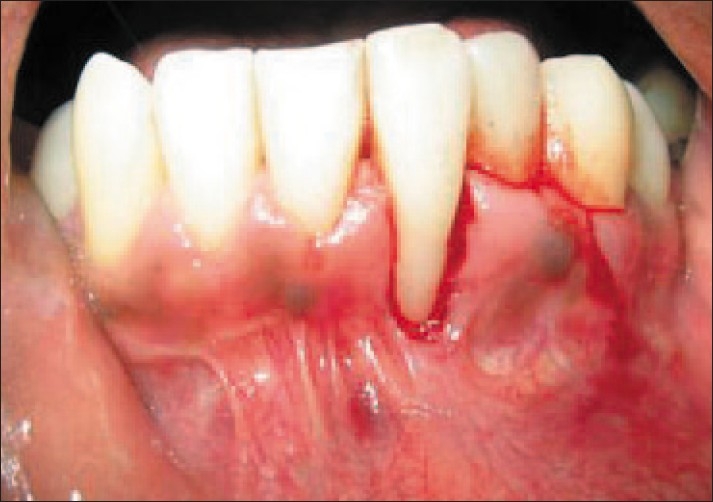
De-epithelialized

**Figure 5:**
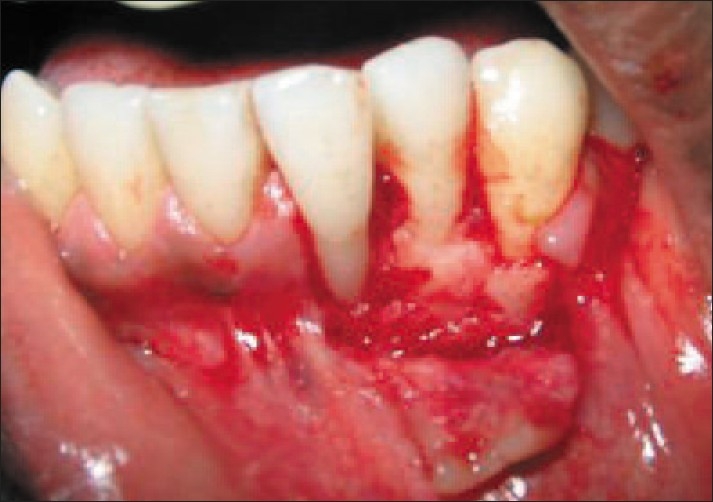
Flap elevation

**Figure 6:**
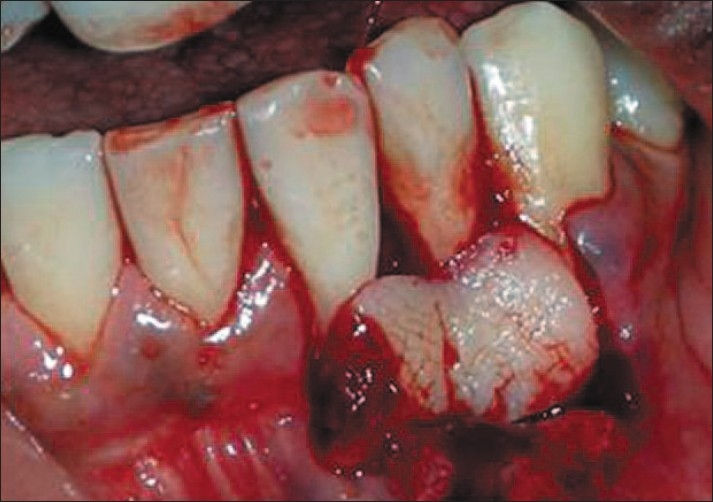
Platelet-rich-fibrin membrane placed

**Figure 7:**
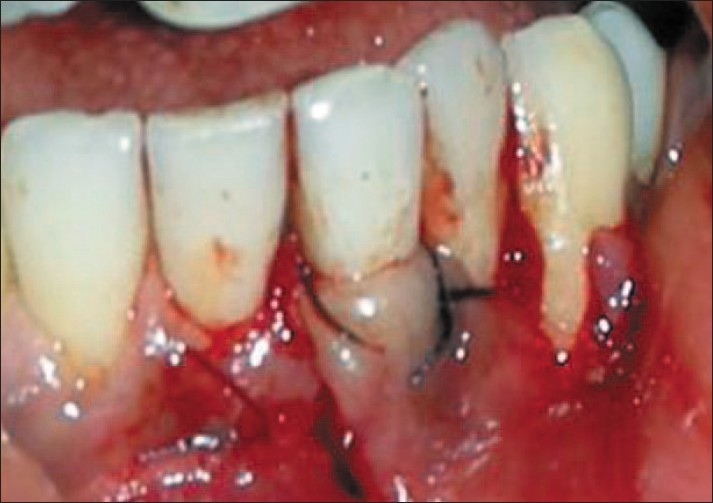
Sutures placed

After proper isolation of the surgical field, the operative sites were anaesthetized using two per cent xylocainehydrochloride with adrenaline (*1:200000*). A reverse bevel ‘V’ shape incision was made along the soft tissue margin of the recipient site in order to remove the epithelium around the root surface [Figure 4]. Then the donor site was prepared by giving a vertical incision from the gingival margin to outline the flap adjacent to the recipient site and a full thickness flap was elevated [Figure 5]. The PRF membrane was placed over the denuded roots and stabilized [Figure 6]. The flap was then slided to completely cover the membrane and secured using sling sutures [Figure 7].

#### Post operative care:

**Figure 8:**
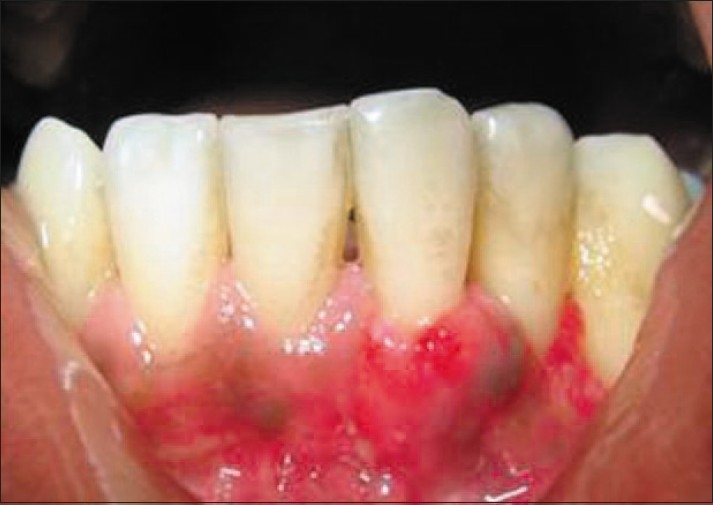
10 days post-operative

Patients were placed on 0.12% chlorhexidine digluconate mouthrinse for four weeks. Systemic antibiotics were prescribed and advised to follow routine post-operative periodontal mucogingival instructions, with minor modifications. They were warned to avoid pulling on their lips to observe the surgical site. The surgical site was repacked after 1 week. Both dressings and sutures were removed 10 days after surgery [Figure 8].

#### Healing:

**Figure 9:**
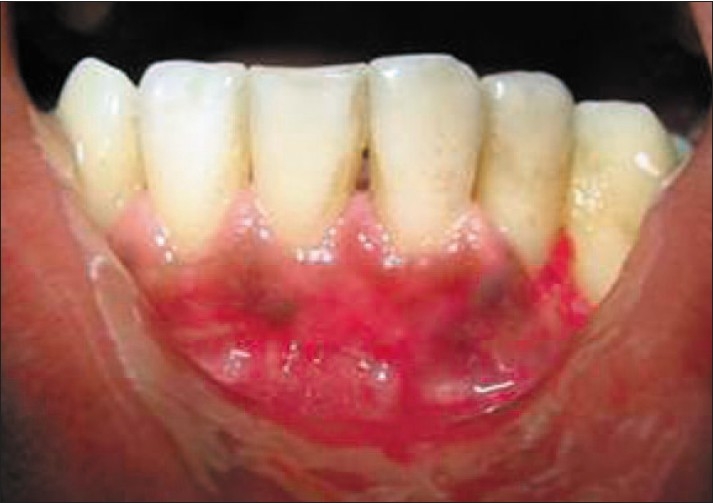
One month post-operative

**Figure 10:**
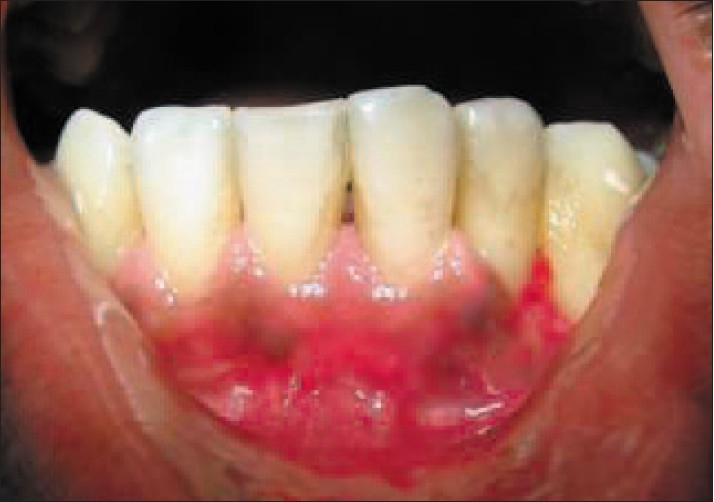
Six months post-operative

Post-operative follow up was done for one month [Figure 9]. In this case, there was no post-operative complication and healing was satisfactory. The patients did not have any post-operative morbidity. Complete coverage was achieved six months after the procedure, with excellent tissue contour and color [Figure 10].

## DISCUSSION

The ultimate goal of any therapeutic intervention aimed at root coverage should be to restore the tissue margin at the cementoenamel junction (CEJ) and to achieve an attachment of the tissues to the root surface so that a normal healthy gingival sulcus with no bleeding on probing and a minimal probing depth is present.[8] Patients have, recently, become more conscious of dental esthetics and accordingly have been demanding precision treatment of their exposed root surfaces.

Various surgical procedures have been described to treat gingival recessions, but these have been demonstrated to heal with a long junctional epithelium, and regeneration has been observed only in the most apical portion of the lesion. Although the bilaminar technique using subepithelial connective tissue grafts still holds the most promising results in root coverage, histological studies show unpredictable healing. The use of PRF membrane in our case report to attain root coverage may alleviate the need for donor site procurement of connective tissue. This has encouraged investigations of a more regenerative nature. The use of enamel matrix protein is one trend aiming at periodontal regeneration and root coverage.[9] The use of barrier membranes is another trend.[10]

In general, a recent innovation in dentistry has been the preparation and use of platelet-rich fibrin (PRF), a concentrated suspension of the growth factors found in platelets. These growth factors are involved in wound healing and postulated as promoters of tissue regeneration. It is both nontoxic and nonimmunoreactive.[11] Early studies have focused on PRP application to bone graft material, showing that it leads to earlier bone regeneration and soft tissue healing.[12] PRP can also be infused into resorbable barrier membranes to retard epithelial migration, as well as to provide localized growth factors to accelerate hard and soft tissue maturation.[13]

PRP may be obtained from autologous blood by the use of plasmaphoresis. PRF was first developed in France by Choukroun *et al*.[14] This second generation platelet concentrate eliminated the risk associated with the use of bovine thrombin. Placement of PRF membrane in recession defects can be used to restore the functional properties of the labial gingiva of the mandibular anterior teeth by repairing gingival defects and re-establishing the continuity and integrity of the zone of keratinized gingiva.

A report of clinical trails comparing the growth factors content of PRF and PRP was presented by Dohan and Diss at the second international Symposium on growth factors held in May 2005.[15] Combining the growth factors has been shown to accelerate bone repair and promote fibroblast proliferation, and increase tissue vascularity, rate of collagen formation, mitosis of mesenchymal stem cells and endothelial cells, as well as osteoblasts, playing key roles in the rate and extent of bone formation. This activity, together with increased vessel ingrowth, is mediated by PDGF and TGF. Because of all of these powerful effects on tissue regeneration, a growing number of human clinical studies have detailed the use of growth factors in reconstructive oral and maxillofacial surgery, periodontal surgery, implants, and sinus grafting.[16]

PRF membrane used in this case report has the advantage of the absence of an anticoagulant, blood begins to coagulate as soon as it comes in contact with the glass surface. Therefore, for successful preparation of PRF, speedy blood collection and immediate centrifugation, before the clotting cascade is initiated, is absolutely essential.

## CONCLUSION

Soft tissue maintenance is the primary line of defense in protecting the tissue from bacterial infection. Although the growth factors and the mechanisms involved are still poorly understood, the ease of applying PRF in the dental clinic and its beneficial outcomes, including reduction of bleeding and rapid healing, holds promise for further procedures. More well designed and properly controlled studies are needed to provide solid evidence of PRF's capacity for and impact on wound healing, soft-tissue reconstruction and (in combination with bone grafts) augmentation procedures, especially in periodontal therapy.
